# The Expression of IL-12 Family Members in Patients with Hypertension and Its Association with the Occurrence of Carotid Atherosclerosis

**DOI:** 10.1155/2020/2369279

**Published:** 2020-04-06

**Authors:** Jing Ye, Yuan Wang, Zhen Wang, Ling Liu, Zicong Yang, Menglong Wang, Yao Xu, Di Ye, Jishou Zhang, Qi Zhou, Yingzhong Lin, Qingwei Ji, Jun Wan

**Affiliations:** ^1^Department of Cardiology, Renmin Hospital of Wuhan University, Cardiovascular Research Institute, Wuhan University, Hubei Key Laboratory of Cardiology, Wuhan 430060, China; ^2^Department of Thyroid Breast Surgery, Renmin Hospital of Wuhan University, Wuhan 430060, China; ^3^Department of Cardiology, The People's Hospital of Guangxi Zhuang Autonomous Region, Nanning 530021, China; ^4^Department of Cardiology, Beijing Anzhen Hospital, Capital Medical University, Beijing Institute of Heart, Lung, and Blood Vessel Diseases, The Key Laboratory of Remodeling-Related Cardiovascular Disease, Ministry of Education, Beijing 100029, China

## Abstract

**Background:**

The interleukin-12 (IL-12) family consists of four members, namely, IL-12, IL-23, IL-27, and IL-35. The aim of this study was to examine the expression of circulating IL-12, IL-23, IL-27, and IL-35 in hypertensive patients.

**Methods:**

Blood samples were collected from hypertensive patients and nonhypertensive (control) subjects, and protein multifactorial monitor kits were used to measure the plasma IL-12, IL-23, IL-27, and IL-35 levels in each sample. In addition, all enrolled subjects underwent ambulatory blood pressure monitoring (ABPM) and vascular stiffness.

**Results:**

Hypertensive patients exhibited higher IL-12, IL-23, and IL-27 levels and lower IL-35 levels than control subjects; IL-12, IL-23, and IL-27 levels were positively correlated with both systolic blood pressure (SBP) and diastolic blood pressure (DBP), while IL-35 levels were negatively correlated with SBP and DBP. IL-12, IL-23, and IL-27 levels gradually increased in patients with grade I, II, and III hypertension, while IL-35 levels gradually reduced. According to the ABPM results, hypertensive patients were divided into the dipper and nondipper hypertension groups; IL-12, IL-23, IL-27, and IL-35 levels showed no differences between the two groups, but IL-12, IL-23, and IL-27 levels in both groups increased compared with those in the control group, while IL-35 levels decreased. Additionally, the expression of these IL-12 family members was influenced by many clinical factors and was independently associated with the occurrence of carotid atherosclerotic plaques.

**Conclusions:**

The changes in IL-12, IL-23, IL-27, and IL-35 levels were not associated with the presence of the nondipper type but were closely associated with the development of carotid atherosclerotic plaque in hypertensive patients.

## 1. Introduction

Among many chronic diseases, the number of patients with hypertension ranks the first in the world; its incidence is gradually increasing, and the affected population is gradually getting younger [1]. Evidence from animal studies and clinical experiments has confirmed that inflammatory response is closely associated with the occurrence of hypertension and the injury of target organs [2–4]. Interleukin (IL) is an important inflammatory factor and may be involved in a variety of inflammatory diseases, including hypertension [2–4].

Unlike other members of the other IL families, the IL-12 family members, namely, IL-12, IL-23, IL-27, and IL-35, are unique, and they all consist of heterogeneous dimers composed of two subunits with the same structural homology [5]. The IL-12 family members can bind to receptors on target organs, activate downstream Janus kinase 1/2-signal transducers and activators of transcription (JAK1/2-STAT1/3/4) pathways, regulate inflammation, and participate in downstream signal regulation [5–7]. Despite their similarities in structure, composition, receptors, and signaling pathways, their biological effects are quite different. Among them, IL-12, IL-23, and IL-27 are considered to be proinflammatory factors, which are mainly secreted by macrophages and effector T lymphocytes, and can participate in various diseases by amplifying inflammatory effects [8, 9]. On the other hand, IL-35, an anti-inflammatory factor secreted mainly by regulatory T cells (Tregs), can protect against tissue damage induced by inflammatory effects [5, 10]. However, recent studies have unexpectedly found that IL-12 can play an anti-inflammatory role in a specific inflammatory microenvironment [11, 12].

A growing evidence suggests that the IL-12 family members are also closely associated with cardiovascular diseases. Clinical data indicated that expression levels of the IL-12 family members were altered in atherosclerosis, coronary artery disease, aortic dissection, atrial fibrillation, viral myocarditis, and cardiomyopathy [11, 13]. Animal studies have showed that the IL-12 family members are involved in the regulation of atherosclerosis mediated by a high-fat diet, ischemic cardiomyopathy mediated by coronary ligation, hypertension, myocardial fibrosis and atrial fibrillation by angiotensin infusion, and viral myocarditis mediated by Coxsackievirus B3 [13–21]. Although the association between the IL-12 family members and hypertension remains unknown, this study is aimed at detecting the levels of circulating IL-12, IL-23, IL-27, and IL-35 in hypertensive patients.

## 2. Methods

### 2.1. Study Population

From February 2018 to February 2019, hypertensive patients who were hospitalized at the Renmin Hospital of Wuhan University, East Hospital of Wuhan University, and the People's Hospital of Guangxi Zhuang Autonomous Region were enrolled into this study. Nonhypertensive (control) subjects who were admitted to the above hospitals for physical examination were used as control subjects. Exclusion criteria were based on previous literature reports, including coronary heart disease, valvular disease, chronic heart failure, secondary hypertension, chronic renal failure, advanced liver disease, and sepsis [22]. Finally, 75 control subjects and 430 hypertensive patients were included in the study. The patients with hypertension were further divided into grade I (*n* = 234), grade II (*n* = 126), and grade III (*n* = 70) groups according to the SBP and/or DBP value of each patient. Written informed consent was signed by the patients themselves or by their family members, and the study was approved by the ethics committees of the People's Hospital of Guangxi Zhuang Autonomous Region (approval no. 20180118EH-L).

### 2.2. Sample Collections and Circulating IL-12, IL-23, IL-27, and IL-35 Detection

Blood samples from all subjects were collected on the morning after admission by clinically experienced nurses. Briefly, fasting blood was collected into blood collection tubes with negative pressure and quickly transferred to the laboratory for centrifugation at 5000 × *g* for 10 min. The supernatant was then collected and stored in an ultra-low temperature refrigerator at -80°C until detection begun.

Frozen plasma samples were taken out from this refrigerator and thawed in a 4°C refrigerator. The protein multifactorial monitor kits (Milliplex, Catalog ID: HCYP4MAG-64K-02.Hum for the detection of IL-12, IL-23, and IL-27 and Catalog ID: HTH17MAG-14K-03.Hum for the measurement of IL-35 and IL-37) were taken out from a 4°C refrigerator and returned to room temperature, and then, plasma IL-12, IL-23, IL-27, and IL-35 concentrations were measured according to the instructions of the manufacturer. The method of liquid-phase chip technology was used to detect OD value per well of each cytokine and calculate cytokine concentration. All assays were performed in duplicate.

### 2.3. Neck Vascular Ultrasound, Ambulatory Blood Pressure Monitoring, and Vascular Stiffness Examination

All the control subjects and hypertensive patients underwent ambulatory blood pressure monitoring (ABPM), neck vascular ultrasound, and vascular stiffness examination; all procedures and analysis of results were performed by doctors with more than 10 years of experience. Neck ultrasound was used to determine whether bilateral carotid arteries were forming carotid atherosclerotic plaques (CAP) in each subject. ABPM was performed to determine dipper and nondipper hypertensions in hypertensive patients, and data on the average 24 h systolic blood pressure (A24h-SBP) and the average 24 h diastolic blood pressure (A24h-DBP) in each patient was collected. In addition, the vascular stiffness examination was conducted, and the results of the brachial-ankle pulse wave velocity (baPWV) and the ankle brachial index (ABI) of each patient's left and right sides were obtained.

### 2.4. Statistical Analysis

All data in this study are represented as mean ± standard deviation or counts (percentage) and analyzed using the SPSS 26.0 statistical software. For data in a continuous variable, the Student's *t*-test and one-way ANOVA were used followed by the Newman-Keuls post hoc test to compare the differences between two or more groups. For data with discontinuous variables, the chi-square test was performed to compare the differences. The correlations between SBP, DBP, baPWV-right, baPWV-left, ABI-right, ABI-left, A24h-SBP, and A24h-DBP and plasma IL-12, IL-23, IL-27, and IL-35 levels in hypertensive patients were analyzed using Spearman's correlation analysis. In addition, the association between plasma IL-12, IL-23, IL-27, and IL-35 levels and the presence of CAP was assessed using univariate analysis and subsequently multivariate linear regression analysis. In this study, the *P* values < 0.05 were considered statistically significant differences.

## 3. Results

### 3.1. Plasma IL-12, IL-23, and IL-27 Levels Were Increased While IL-35 Levels Were Decreased in Hypertensive Patients

Compared with the control group, the hypertension group showed more overweight (BMI ≥ 25 kg/m^2^), type 2 diabetes mellitus (T2DM), hyperlipidemia (HLP), sleep apnea hypopnea syndrome (SAHS), hypoxemia, a higher frequency of CAP patients, higher body mass index (BMI), SBP, DBP, fasting glucose (Glu), insulin, total triglycerides (TG), low-density lipoprotein cholesterol (LDL-C), creatinine (CREA), uric acid (UA), creatine kinase myocardial band (CK-MB), C-reactive protein (CRP), plasma total homocysteine (tHcy) levels, lower hemoglobin a1c (HbAlc), and high-density lipoprotein cholesterol (HDL-C) levels. No differences in the frequency of male and elderly patients, smokers, and patients showing alcohol consumption, age, heart rate (HR), and total cholesterol levels were observed between these two groups. The clinical characteristics of each group are listed in Table 1.

**Figure 1:**
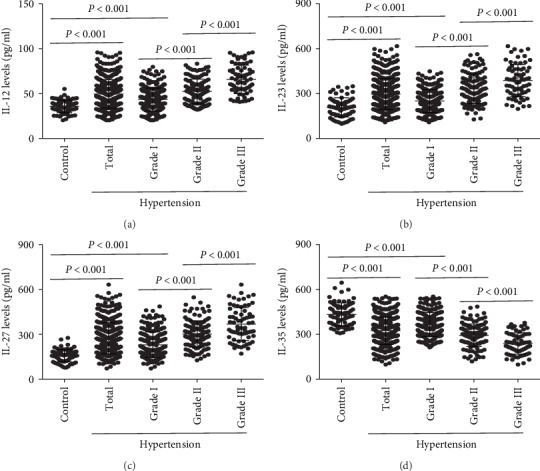
The expression of IL-12 family members in hypertensive patients. The plasma (a) IL-12, (b) IL-23, (c) IL-27, and (d) IL-35 levels in control subjects, hypertension group, grade I group, grade II group, and grade III group were detected by protein multifactor monitoring kits.

In addition, the plasma IL-12, IL-23, and IL-27 levels were all increased, and the plasma IL-35 levels were decreased in patients with hypertension, compared with control subjects (Figures 1(a)–1(d)). The IL-12, IL-23, and IL-27 levels also gradually increased in patients with grade I, II, and III hypertension, and those in all groups were higher than those in the control group (Figures 1(a)–1(c)). However, IL-35 exhibited an opposite trend compared to that shown by the IL-12, IL-23, and IL-27 levels in patients with grade I, II, and III hypertension and those from the control group (Figure 1(d)). The plasma IL-12, IL-23, IL-27, and IL-35 levels in each group are listed in Table 2.

**Figure 2:**
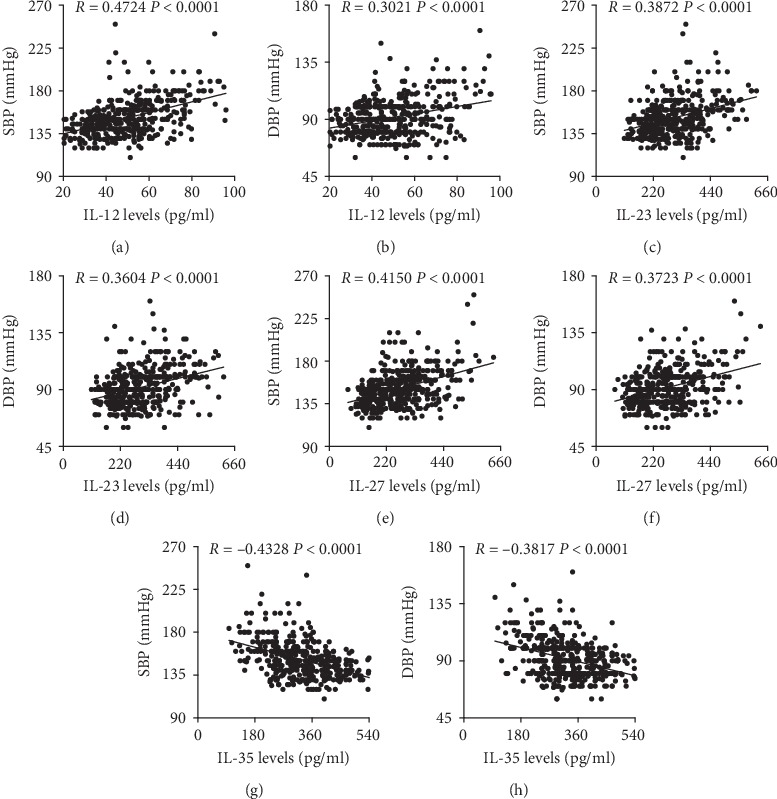
The correlations between plasma IL-12 family members and blood pressure. The correlations between SBP and DBP and plasma (a, b) IL-12, (c, d) IL-23, (e, f) IL-27, and (g, h) IL-35 were analyzed using Spearman's correlation analysis.

Furthermore, the plasma IL-12, IL-23, and IL-27 levels were all positively correlated with both the SBP and DBP in hypertensive patients (Figures 2(a)–2(f)), while plasma IL-35 levels exhibited negative correlation with both SBP and DBP (Figures 2(g) and 2(h)).

### 3.2. Correlation between the Results of Vascular Stiffness Examination and ABPM and Plasma IL-12 Family Members

For the results of vascular stiffness examination and ABPM, higher baPWV-right, baPWV-left, ABI-right, ABI-left, A24h-SBP, and A24h-DBP were observed in hypertensive patients. All the results in each group are listed in Table 3.

Spearman's correlation analysis showed that plasma IL-12, IL-23, and IL-27 levels were all positively correlated with baPWV-right, baPWV-left, A24h-SBP, and A24h-DBP while they were negatively correlated with ABI-right and ABI-left, whereas IL-35 exhibited opposite trends to IL-12, IL-23, and IL-37. The *R* values and *P* values of each interleukin are listed in Table 4.

### 3.3. Nondipper Hypertension Did Not Affect the Expression of These Cytokines

The hypertensive patients were divided into the dipper group (*n* = 221) and nondipper group (*n* = 209) according to the results of ABPM. The nondipper group exhibited more CAP presence; no differences in other clinical characteristics were observed. The clinical characteristics of each group are listed in Table 5.

**Figure 3:**
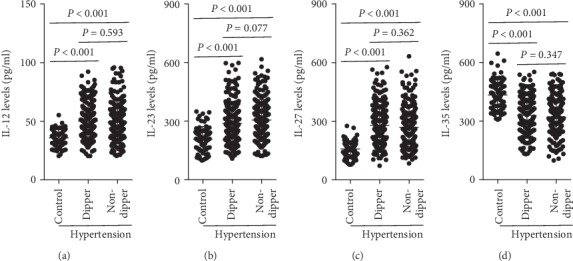
Expression of the IL-12 family members in dipper hypertension and nondipper hypertension. Plasma (a) IL-12, (b) IL-23, (c) IL-27, and (d) IL-35 levels in control subjects, dipper hypertensive patients, and nondipper hypertensive patients were measured.

The results showed no significant differences in plasma IL-12 levels between the dipper and the nondipper groups, while the plasma IL-12 levels in both these groups were higher than those in the control group (Figure 3(a)). The plasma IL-23 and IL-27 levels exhibited similar trends compared to that of IL-12 (Figures 3(b) and 3(c)). No significant differences were observed in the IL-35 levels between these two groups, while both were significantly lower than those in the control group (Figure 3(d)). The expression of the plasma IL-12 family members is listed in Table 6.

### 3.4. Effects of Clinical Characteristics on the Expression of Plasma IL-12 Members in Hypertensive Patients

The hypertensive patients were divided into two groups based on the following factors: male gender, elderly, overweight, smoking, drinking, T2DM, HLP, SAHS, hypoxemia, and CAP. The results showed that elderly patients with hypertension who were overweight, had T2DM, SAHS, hypoxemia, or CAP, and were smokers showed higher plasma IL-12 levels when compared with those that did not show these characteristics. On the other hand, smoking, T2DM, HLP, SAHS, hypoxemia, and CAP exhibited higher plasma IL-23 expression in hypertensive patients. The plasma IL-27 levels in hypertensive patients were upregulated by overweight, smoking, drinking, T2DM, HLP, SAHS, hypoxemia, and CAP. Furthermore, male and elderly hypertensive patients who were smokers and had T2DM, SAHS, and CAP showed lower plasma IL-35 levels than those without these characteristics. The sample sizes and plasma interleukin levels in each group are listed in Table 7.

### 3.5. Plasma IL-12, IL-23, IL-27, and IL-35 Levels Were All Closely Associated with the Presence of CAP

The clinical characteristics, including male gender, elderly, overweight, smoking, drinking, nondipper, T2DM, HLP, SAHS, hypoxemia, CRP, tHcy, and the IL-12 members, were used to perform univariate analysis, and the results showed that IL-12, IL-23, IL-27, IL-35, male gender, elderly, overweight, drinking, nondipper, T2DM, HLP, SAHS, hypoxemia, and tHcy showed a trend. Then, the variables that showed the trend mentioned above were used to further perform multivariate regression analysis. The results showed that plasma IL-12, IL-23, and IL-27 levels were all independently positively associated with the occurrence of CAP, while plasma IL-35 levels were independently negatively associated with the presence of CAP. The *β* value, 95% CI of *β*, and *P* values are listed in Table 8.

## 4. Discussion

In this study, the levels of circulating IL-12, IL-23, IL-27, and IL-35 were measured in hypertensive patients. The results showed that plasma expression levels of proinflammatory cytokines IL-12, IL-23, and IL-27 were increased in hypertensive patients and positively correlated with their blood pressures. On the other hand, the circulating levels of the anti-inflammatory cytokine IL-35 were decreased in hypertensive patients and negatively correlated with blood pressure. In addition, the presence of nondipper hypertension was independently positively correlated with the occurrence of carotid atherosclerosis, although it did not affect the expression of the IL-12 family members. The expression of these inflammatory cytokines in hypertensive patients was also affected by various factors. Furthermore, IL-12, IL-23, and IL-27 were all independently positively correlated with the onset of CAP, while IL-35 was independently negatively correlated with the occurrence of CAP.

It is well known that hypertension is a moderate chronic inflammatory disease. Infiltration of immune and inflammatory cells, including macrophages, lymphocytes, and dendritic cells, can be observed at all stages of hypertension development [11, 23, 24]. Proinflammatory cytokines do not only promote the differentiation or maturation of these cells but also upregulate the release of a variety of inflammatory substances, thereby inducing inflammatory effects, which further increases the blood pressure and aggravates target organ injury [25, 26]. There is a growing evidence from animal and clinical studies that blocking proinflammatory signals or upregulating anti-inflammatory cytokines can significantly lower blood pressure and reduce organ injury [25, 26]. These evidences may suggest that the occurrence of hypertension and its mediated target organ injury may be a combination of proinflammatory roles and anti-inflammatory effects imbalances. In this study, the expression of IL-12 family members was detected, and the results showed that the expression levels of these cytokines were significantly changed in hypertensive patients compared with those in the control group. The expression levels of proinflammatory factors IL-12, IL-23, and IL-27 were increased significantly, while the expression of anti-inflammatory factor IL-35 was decreased, and these results partially support the above conjecture. The expression levels of a variety of other inflammatory markers also changed in hypertensive patients, representative ones are TNF-*α* and IL-6, suggesting that the occurrence of hypertension may be the result of the combined action of a variety of inflammatory factors.

Interestingly, in a recent study, we unexpectedly found that treatment with the proinflammatory cytokine IL-12, rather than the anti-inflammatory cytokine IL-35, inhibited Ang II-induced M1 macrophage differentiation, significantly reduced local vascular inflammation, and reduced blood pressure [11]. In fact, proinflammatory factors do not amplify the inflammatory effects in all inflammatory responses but are closely related to the collective inflammatory microenvironment induced by specific inflammatory substances. For example, IL-22, also a proinflammatory factor, has also been observed to have anti-inflammatory effects, and as an anti-inflammatory cytokine, IL-37 amplifies the inflammatory response in larger doses [27, 28]. These findings may also shed some light on the complexity of the link between inflammation and hypertension. Moreover, in the course of hypertension and related target organ injury, we may consider the role of multiple inflammatory factors as a whole, and there are several limitations in studying the role of a single inflammatory factor in hypertension.

Nondipper hypertension is a special type of hypertension, which accounts for a small proportion, but its harm is significantly higher than that of dipper hypertension. A large number of clinical studies have shown that compared with the dipper hypertensive patients, the nondipper hypertensive patients are more likely to have cardiac hypertrophy, decreased cardiac function, kidney injury, stroke, and all-cause mortality [29–31]. In this study, the expression of circulating IL-12 family members in the nondipper hypertensive patients was analyzed, and the results showed that there was no significant difference in plasma IL-12, IL-23, IL-27, and IL-35 levels between the dipper and nondipper hypertension groups. Although the special mechanisms of the occurrence of dipper hypertension are not fully understood, there is evidence suggesting that it may be significantly associated with a decrease in nocturnal sympathetic excitability or even an increase in abnormal activity, as well as a decrease in related neurotransmitters [32]. These results suggest that a variety of factors may affect IL-12 family members, but not sympathetic excitability.

Previous studies have confirmed that the expression of interleukin family members is regulated by a variety of pathological factors, such as oxidative stress and calcium overload, especially the inflammatory response [5]. In order to determine whether overweight, smoking, T2DM, and other factors that are associated with inflammation affected the expression of the IL-12 family members in hypertensive patients, all the patients were divided into two groups according to whether they fulfilled these criteria or not. The results showed that the plasma expression of each IL-12 family member was affected by many factors. The results suggest that different IL-12 family members may be involved in different inflammatory processes and that one or more IL-12 family members may be involved in the same inflammatory environment. In addition, animal studies have demonstrated that all members of the IL-12 family are involved in the development of high-fat-diet-induced atherosclerosis, with IL-12, IL-23, and IL-27 promoting the process and IL-35 inhibiting it [14, 15, 18]. In our clinical study, the results of multiple regression analysis also showed that all members of the IL-12 family were associated with the occurrence of CAP. Our study further complements the close association between IL-12 family members and arterial plaque disease, despite the fact that the inflammatory environment in animals is different from that in humans.

In conclusion, our present study found for the first time that the expression of all the plasma IL-12 family members showed significant changes in hypertensive patients and was independently associated with the occurrence of CAP, although the change in trends in hypertension and their role in the presence of CAP may not be consistent. Nevertheless, our study had some limitations. First, we did not observe the dynamic changes of these IL-12 family members. Second, we did not follow up patients to observe the incidence of long-term complications and all-cause mortality in these patients.

## Figures and Tables

**Table 1:** Clinical characteristics of hypertensive patients and control subjects.

Characteristics	Control	Hypertension			
		Total	Grade I	Grade II	Grade III
Male (*n*, %)	37 (49.3)	241 (56.0)	124 (53.0)	76 (60.3)	41 (58.6)
Elderly (*n*, %)	18 (24.0)	151 (35.1)	89 (38.0)	49 (38.9)	13 (18.6)^#&$^
Overweight (*n*, %)	17 (22.7)	281 (65.3)^∗^	144 (61.5)^∗^	88 (69.8)^∗^	49 (70.0)^∗^
Smoking (*n*, %)	23 (30.7)	155 (36.0)	79 (33.8)	52 (41.3)	24 (34.3)
Drinking (*n*, %)	13 (17.3)	110 (25.6)	54 (23.1)	39 (31.0)	17 (24.3)
Dipper (*n*, %)	—	209 (48.6)	111 (47.4)	65 (51.6)	33 (47.1)
T2DM (*n*, %)	5 (6.7)	114 (26.5)^∗^	58 (24.8)^∗^	40 (31.7)^∗^	16 (22.9)^∗^
HLP (*n*, %)	17 (22.6)	241 (56.0)^∗^	123 (52.6)^∗^	61 (48.4)^∗^	36 (51.4)^∗^
ASHS (*n*, %)	0 (0.0)	52 (12.1)^∗^	26 (11.1)^∗^	21 (16.7)^∗^	5 (7.1)
Hypoxemia (*n*, %)	0 (0.0)	49 (11.4)^∗^	26 (11.1)^∗^	20 (15.9)^∗^	3 (4.3)
CAP (*n*, %)	0 (0.0)	153 (35.6)^∗^	65 (27.8)^∗^	50 (39.7)^∗^	38 (54.3)^∗^^&^
Age (years)	53 ± 11	51 ± 15	53 ± 15	52 ± 15	46 ± 14^∗^^#&$^
BMI (kg/m^2^)	23 ± 4	27 ± 4^∗^	26 ± 4^∗^^#^	27 ± 4^∗^^&^	27 ± 4^∗^
HR (bpm)	73 ± 11	73 ± 12	71 ± 10^#^	74 ± 11^&^	77 ± 15^#&^
SBP (mmHg)	115 ± 14	151 ± 19^∗^	140 ± 10^∗^^#^	158 ± 10^∗^^#&^	179 ± 20^∗^^#&$^
DBP (mmHg)	75 ± 9	91 ± 15^∗^	83 ± 9^∗^^#^	96 ± 9^∗^^#&^	112 ± 16^∗^^#&$^
Glu (mmol/L)	4.7 ± 0.8	5.9 ± 1.5^∗^	5.8 ± 1.5^∗^	5.9 ± 1.5^∗^	6.0 ± 1.5^∗^
HbAlc (%)	4.6 ± 1.0	5.9 ± 1.0^∗^	5.8 ± 0.9^∗^	5.9 ± 1.0^∗^	5.9 ± 1.1^∗^
Insulin (*μ*U/mL)	6.7 ± 4.4	9.4 ± 6.9^∗^	9.7 ± 7.3^∗^	9.0 ± 6.5^∗^	8.8 ± 9.3
TC (mmol/L)	4.5 ± 0.7	4.7 ± 1.1	4.6 ± 1.1	4.7 ± 0.9	4.9 ± 1.1^#&^
TG (mmol/L)	1.3 ± 1.0	1.9 ± 1.5^∗^	1.9 ± 1.3^∗^	2.1 ± 2.0^∗^	1.9 ± 1.1^∗^
HDL-C (mmol/L)	1.2 ± 0.3	1.1 ± 0.3	1.1 ± 0.3	1.1 ± 0.3	1.1 ± 0.3
LDL-C (mmol/L)	2.4 ± 0.7	2.8 ± 0.9^∗^	2.8 ± 1.0^∗^	2.8 ± 0.7^∗^	3.1 ± 1.0^∗^^&^
CREA (*μ*mol/L)	62 ± 12	77 ± 23^∗^	75 ± 20^∗^	73 ± 27^∗^	94 ± 24^∗^^#&$^
UA (*μ*mol/L)	344 ± 106	386 ± 114^∗^	383 ± 112^∗^	383 ± 116	403 ± 116^∗^
CK-MB (U/L)	1.12 ± 0.47	1.42 ± 1.12^∗^	1.41 ± 1.28^∗^	1.52 ± 0.97^∗^	1.33 ± 0.67
CRP (mg/L)	0.66 ± 0.72	2.8 ± 4.7^∗^	2.8 ± 4.6^∗^	2.5 ± 3.7^∗^	3.6 ± 6.3^∗^
tHcy (*μ*mol/L)	11.6 ± 6.6	14.7 ± 8.4^∗^	13.9 ± 7.2^∗^	14.7 ± 8.3^∗^	17.3 ± 11.4^∗^^#&^
Medical treatments (*n*, %)					
ACEI/ARB	5 (6.7)	241 (56.0)^∗^	131 (56.0)^∗^	75 (59.5)^∗^	35 (50.0)^∗^
*β*-Blocker	5 (6.7)	181 (42.1)^∗^	101 (41.2)^∗^	49 (38.9)^∗^	31 (44.3)^∗^
CCB	0 (0.0)	297 (69.1)^∗^	161 (68.8)^∗^	85 (67.5)^∗^	51 (72.9)^∗^
Diuretics	0 (0.0)	126 (29.3)^∗^	62 (26.5)^∗^	45 (35.7)^∗^	19 (27.1)^∗^
*α*-Blocker	2 (2.6)	27 (6.3)	12 (5.1)	9 (7.1)	6 (8.6)

T2DM: type 2 diabetes mellitus; HLP: hyperlipidemia; ASHS: sleep apnea hypopnea syndrome; CAP: carotid atherosclerotic plaque; BMI: body mass index; HR: heart rate; SBP: systolic blood pressure; DBP: diastolic blood pressure; Glu: fasting glucose; HbAlc: hemoglobin a1c; TC: total cholesterol; TG: total triglycerides; HDL-C: high-density lipoprotein cholesterol; LDL-C: low-density lipoprotein cholesterol; CREA: creatinine; UA: uric acid; CK-MB: creatine kinase myocardial band; CRP: C-reactive protein; tHcy: plasma total homocysteine; ACEI: angiotensin-converting enzyme inhibitor; ARB: angiotensin receptor blocker; CCB: calcium channel blocker. ^∗^*P* < 0 05 vs. control group; ^#^*P* < 0 05 vs. total group; ^&^*P* < 0 05 vs. grade I group; ^$^*P* < 0 05 vs. grade II group.

**Table 2:** Interleukins in each group.

Interleukins	Control	Hypertension			
		Total	Grade I	Grade II	Grade III
IL-12	35.9 ± 7.0	49.7 ± 15.8^∗^	43.5 ± 12.8^∗^^#^	52.4 ± 13.7^∗^^#&^	65.8 ± 15.9^∗^^#&$^
IL-23	208 ± 64	297 ± 103^∗^	251 ± 76^∗^^#^	331 ± 99^∗^^#&^	388 ± 107^∗^^#&$^
IL-27	158 ± 41	274 ± 104^∗^	231 ± 85^∗^^#^	303 ± 88^∗^^#&^	368 ± 110^∗^^#&$^
IL-35	433 ± 77	325 ± 92^∗^	367 ± 81^∗^^#^	294 ± 75^∗^^#&^	239 ± 71^∗^^#&$^

^∗^ *P* < 0.05 vs. control group; ^#^*P* < 0.05 vs. total group; ^&^*P* < 0.05 vs. grade I group; ^$^*P* < 0.05 vs. grade II group.

**Table 3:** Results of vascular stiffness examination and ambulatory blood pressure monitoring in each group.

Examination	Characteristics	Control	Hypertension			
			Total	Grade I	Grade II	Grade III
Vascular stiffness	baPWV-right	1295 ± 109	1538 ± 189^∗^	1467 ± 165^∗^^#^	1594 ± 173^∗^^#&^	1673 ± 184^∗^^#&$^
	baPWV-left	1317 ± 91	1545 ± 212^∗^	1482 ± 208^∗^^#^	1590 ± 177^∗^^#&^	1677 ± 206^∗^^#&$^
	ABI-right	1.27 ± 0.09	1.08 ± 0.12^∗^	1.14 ± 0.12^∗^^#^	1.05 ± 0.08^∗^^#&^	0.97 ± 0.06^∗^^#&$^
	ABI-left	1.26 ± 0.11	1.08 ± 0.11^∗^	1.12 ± 0.10^∗^^#^	1.06 ± 0.07^∗^^#&^	0.97 ± 0.07^∗^^#&$^

ABPM	A24h-SBP	116 ± 9	146 ± 12^∗^	140 ± 9^∗^^#^	149 ± 11^∗^^#&^	159 ± 10^∗^^#&$^
	A24h-DBP	75 ± 6	88 ± 12^∗^	83 ± 9^∗^^#^	89 ± 13^∗^^#&^	100 ± 11^∗^^#&$^

baPWV: brachial-ankle pulse wave velocity; ABI: ankle brachial index; A24h-SBP: average 24 h systolic blood pressure; A24h-DBP: average 24 h diastolic blood pressure. ^∗^*P* < 0.05 vs. control group; ^#^*P* < 0.05 vs. total group; ^&^*P* < 0.05 vs. grade I group; ^$^*P* < 0.05 vs. grade II group.

**Table 4:** Correlation between the results of vascular stiffness examination and ABPM and plasma IL-12 family members.

Factors	IL-12		IL-23		IL-27		IL-35	
	*R* value	*P* value	*R* value	*P* value	*R* value	*P* value	*R* value	*P* value
baPWV-right	0.3335	<0.0001	0.2872	<0.0001	0.2463	<0.0001	-0.3635	<0.0001
baPWV-left	0.2022	<0.0001	0.2042	<0.0001	0.2025	<0.0001	-0.3046	<0.0001
ABI-right	-0.2952	<0.0001	-0.2452	<0.0001	-0.2449	<0.0001	0.1924	<0.0001
ABI-left	-0.2841	<0.0001	-0.1912	<0.0001	-0.2461	<0.0001	0.1351	<0.0001
A24h-SBP	0.2414	<0.0001	0.2717	<0.0001	0.2694	<0.0001	-0.2400	0.0050
A24h-DBP	0.2109	<0.0001	0.2764	<0.0001	0.3466	<0.0001	-0.3071	<0.0001

**Table 5:** Clinical characteristics of the dipper group and the nondipper group.

Characteristics	Dipper group	Nondipper group	*P* value
Male (*n*, %)	128 (57.9)	113 (54.1)	0.438
Elderly (*n*, %)	78 (35.3)	73 (34.9)	0.999
Overweight (*n*, %)	145 (65.6)	136 (65.1)	0.919
Smoking (*n*, %)	77 (34.8)	78 (37.3)	0.616
Drinking (*n*, %)	55 (24.9)	55 (26.3)	0.742
T2DM (*n*, %)	52 (23.5)	62 (29.7)	0.157
HLP (*n*, %)	108 (48.9)	112 (53.6)	0.336
ASHS (*n*, %)	24 (10.9)	28 (13.4)	0.461
Hypoxemia (*n*, %)	22 (10.0)	27 (12.9)	0.363
CAP (*n*, %)	60 (27.1)	93 (44.5)	<0.001
Grade I/II/III (*n*)	123/61/37	111/65/33	0.375/0.459/0.796
Age (years)	50 ± 16	53 ± 14	0.105
BMI (kg/m^2^)	27 ± 4	27 ± 4	0.962
HR (bpm)	72 ± 11	73 ± 12	0.522
SBP (mmHg)	151 ± 17	152 ± 20	0.454
DBP (mmHg)	91 ± 14	91 ± 16	0.915
Glu (mmol/L)	5.8 ± 1.4	5.9 ± 1.6	0.553
HbAlc (%)	5.9 ± 0.9	5.9 ± 1.0	0.839
Insulin (*μ*U/mL)	9.3 ± 7.3	9.5 ± 6.5	0.756
TC (mmol/L)	4.7 ± 1.1	4.6 ± 1.0	0.241
TG (mmol/L)	1.9 ± 1.6	2.0 ± 1.5	0.491
HDL-C (mmol/L)	1.1 ± 0.3	1.1 ± 0.3	0.133
LDL-C (mmol/L)	2.9 ± 0.9	2.8 ± 0.9	0.093
CREA (*μ*mol/L)	75 ± 22	80 ± 26	0.206
UA (*μ*mol/L)	384 ± 110	389 ± 118	0.663
CK-MB (U/L)	1.42 ± 0.94	1.43 ± 1.28	0.914
CRP (mg/L)	3.0 ± 5.1	2.7 ± 4.3	0.540
tHcy (*μ*mol/L)	14.1 ± 7.3	15.4 ± 9.4	0.105
Medical treatments (*n*, %)			
ACEI/ARB	123 (55.7)	118 (56.5)	0.845
*β*-Blocker	98 (44.3)	83 (39.7)	0.379
CCB	150 (67.9)	147 (70.3)	0.603
Diuretics	64 (29.0)	62 (29.7)	0.833
*α*-Blocker	14 (6.3)	13 (6.2)	0.999

**Table 6:** Interleukins in the dipper group and the nondipper group.

Interleukins	Control	Hypertension	
		Dipper	Nondipper
IL-12	35.9 ± 7.0	50.1 ± 15.2^∗^	49.3 ± 16.4^∗^
IL-23	208 ± 64	288 ± 101^∗^	306 ± 105^∗^
IL-27	158 ± 41	279 ± 102^∗^	269 ± 107^∗^
IL-35	433 ± 77	329 ± 89^∗^	320 ± 94^∗^

^∗^ *P* < 0.05 vs. control group.

**Table 7:** Interleukin levels in subjects with or without factors.

Factors	Numbers		With factor group vs. without factor group			
	With	Without	IL-12	IL-23	IL-27	IL-35
Male	278	227	49.6 ± 17.1 vs. 49.9 ± 14.6	297 ± 101 vs. 298 ± 104	279 ± 106 vs. 271 ± 103	314 ± 91 vs. 341 ± 93^∗^
Elderly	169	336	53.9 ± 18.5 vs. 47.6 ± 15.7^∗^	291 ± 106 vs. 301 ± 103	269 ± 101 vs. 278 ± 109	294 ± 128 vs. 341 ± 88^∗^
Overweight	298	207	52.9 ± 18.4 vs. 43.4 ± 14.5^∗^	298 ± 105 vs. 294 ± 110	292 ± 116 vs. 244 ± 108^∗^	331 ± 107 vs. 333 ± 108
Smoking	178	327	52.8 ± 21.5 vs. 48.3 ± 17.1^∗^	322 ± 119 vs. 281 ± 109^∗^	303 ± 122 vs. 257 ± 112^∗^	306 ± 114 vs. 336 ± 106^∗^
Drinking	123	382	50.8 ± 19.4 vs. 49.7 ± 17.2	302 ± 104 vs. 294 ± 113	315 ± 137 vs. 259 ± 118^∗^	318 ± 112 vs. 328 ± 109
T2DM	119	386	53.4 ± 19.6 vs. 48.3 ± 16.1^∗^	327 ± 131 vs. 284 ± 108^∗^	316 ± 166 vs. 267 ± 183^∗^	297 ± 122 vs. 331 ± 109^∗^
HLP	258	247	49.8 ± 19.1 vs. 50.2 ± 18.2	317 ± 117 vs. 282 ± 109^∗^	308 ± 176 vs. 256 ± 196^∗^	317 ± 116 vs. 332 ± 112
SAHS	52	453	55.9 ± 19.6 vs. 48.8 ± 19.4^∗^	357 ± 122 vs. 286 ± 118^∗^	335 ± 171 vs. 270 ± 183^∗^	284 ± 120 vs. 334 ± 114^∗^
Hypo	49	456	58.1 ± 22.5 vs. 48.5 ± 30.9^∗^	348 ± 121 vs. 290 ± 119^∗^	358 ± 165 vs. 270 ± 194^∗^	296 ± 119 vs. 330 ± 115
CAP	153	352	59.2 ± 17.6 vs. 44.5 ± 14.3^∗^	342 ± 127 vs. 261 ± 96^∗^	326 ± 140 vs. 244 ± 97^∗^	284 ± 223 vs. 357 ± 108^∗^

^∗^ *P* < 0.05 vs. with factor group.

**Table 8:** Association between serum IL-12, IL-23, IL-27, and IL-35 levels and the presence of CAP was assessed by univariate analysis and subsequent multivariate linear regression analysis.

Variables	Univariate			Multivariate		
	*β*	95% CI	*P* value	*β*	95% CI	*P* value
IL-12	0.343	0.257 to 0.430	<0.001	0.120	0.023 to 0.217	0.016
IL-23	0.417	0.334 to 0.501	<0.001	0.156	0.052 to 0.261	0.003
IL-27	0.370	0.285 to 0.456	<0.001	0.114	0.010 to 0.219	0.032
IL-35	-0.374	-0.459 to -0.289	<0.001	-0.160	-0.254 to -0.066	0.001
Male	0.101	0.009 to 0.192	0.031	0.013	-0.079 to 0.106	0.775
Elderly	0.125	0.034 to 0.216	0.007	0.142	0.062 to 0.222	0.001
Overweight	0.126	0.035 to 0.217	0.007	0.030	-0.053 to 0.112	0.482
Smoking	0.072	-0.020 to 0.163	0.124			
Drinking	0.125	0.034 to 0.216	0.007	0.076	-0.010 to 0.161	0.084
Nondipper	0.208	0.119 to 0.298	<0.000	0.133	0.053 to 0.212	0.001
T2DM	0.121	0.030 to 0.212	0.009	0.019	-0.063 to 0.102	0.644
HLP	0.171	0.080 to 0.261	<0.000	0.074	-0.008 to 0.155	0.076
SAHS	0.171	0.080 to 0.261	<0.000	0.283	0.044 to 0.521	0.020
Hypoxemia	0.130	0.039 to 0.221	0.005	-0.177	-0.414 to 0.061	0.145
CRP	0.025	-0.067 to 0.117	0.597			
tHcy	0.131	0.040 to 0.222	0.005	0.032	-0.053 to 0.118	0.455

## Data Availability

We declare that the materials described in the manuscript, including all relevant raw data, will be freely available to any scientist wishing to use them for noncommercial purposes, without breaching participant confidentiality.
